# Supplementary material for: Freshwater trematodes differ from marine trematodes in patterns connected with division of labor
Source: PeerJ. 2024 Apr 12;12:e17211. doi: 10.7717/peerj.17211 (PMC11017974; doi:10.7717/peerj.17211)
Supplement: Supplemental Information 1 — Two photos of the same redia taken two seconds apart were superimposed using ImageJ’s Merge Channels function (one image red, one image green) and the largest distance moved by any part of the anterior end of the redia was measured in micrometers. [file peerj-12-17211-s001.pdf]

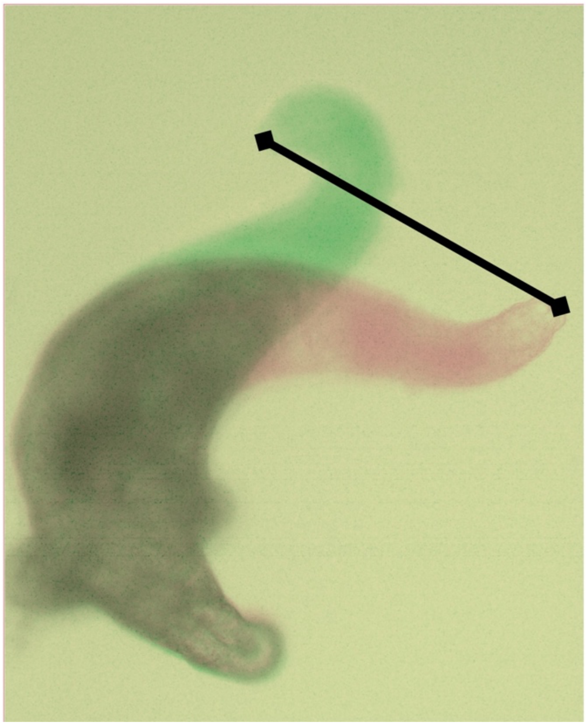

Supplementary File 1: Example image showing how activity measurements were taken. Two photos of the same redia taken two seconds apart were superimposed using ImageJ's Merge Channels function (one image red, one image green) and the largest distance moved by any part of the anterior end of the redia was measured in micrometers.
