# Supplementary material for: Freshwater trematodes differ from marine trematodes in patterns connected with division of labor
Source: PeerJ. 2024 Apr 12;12:e17211. doi: 10.7717/peerj.17211 (PMC11017974; doi:10.7717/peerj.17211)

Supplementary File 2: Representative photos of the snails collected from each family. All snails are shown in the wells of a 12- or 24-well cell culture plate. Snail families are as follows: Top Left: Hydrobiidae; Top Right: Lymnaidae (some lymnaid snails we collected looked more like the pictured physid); Middle Left: Physidae; Middle Right: Planorbidae (some planorbid snails we collected were significantly larger than the one shown); Bottom Left: Valvatidae; Bottom Right: Viviparidae (most collected viviparid snails were too large to fit in the cell culture plate wells and looked more like the inset photo; the main photo is of a very young snail).

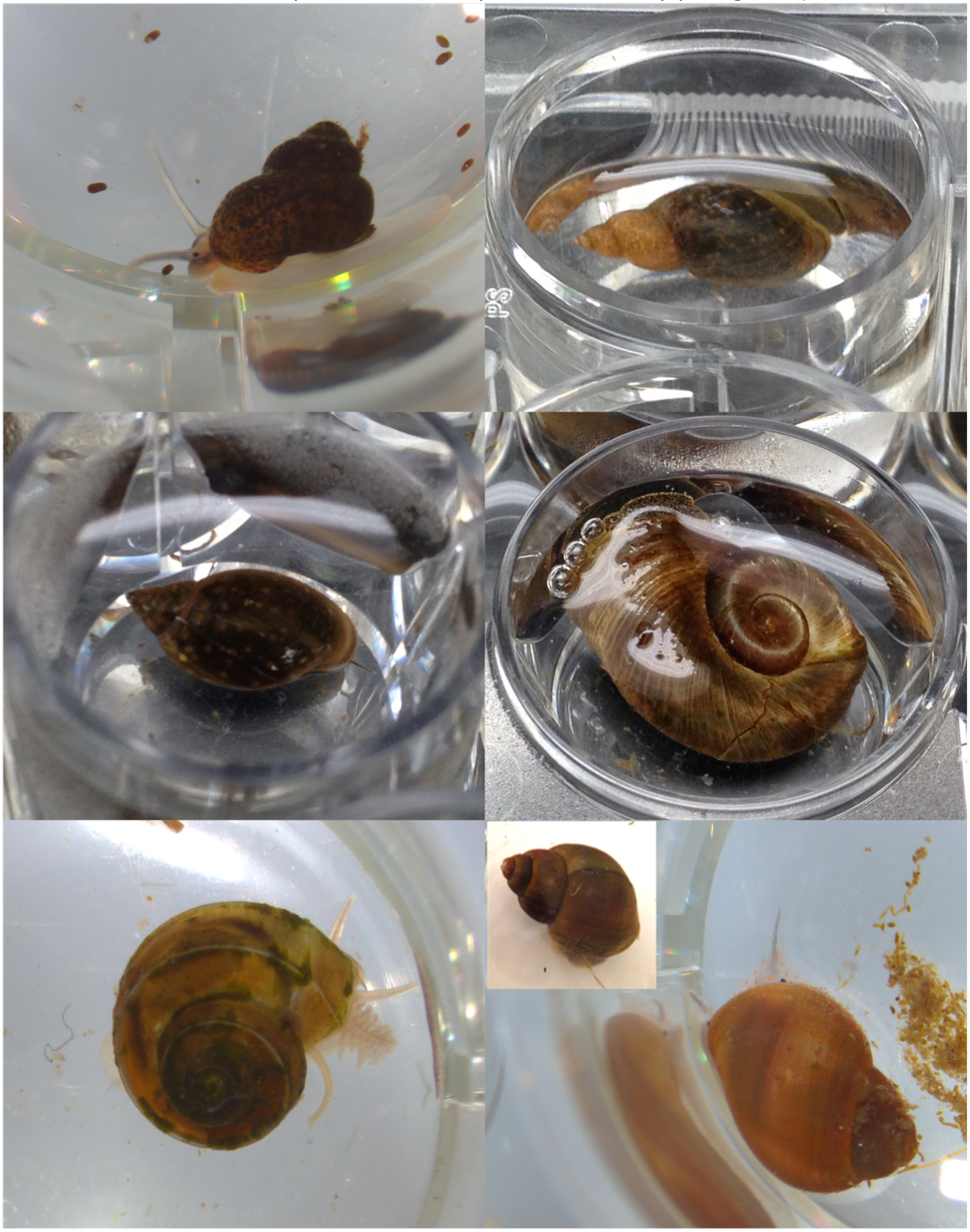

Supplement: Supplemental Information 2 — All snails are shown in the wells of a 12- or 24-well cell culture plate. Snail families are as follows: Top Left: Hydrobiidae; Top Right: Lymnaidae (some lymnaid snails we collected looked more like the pictured physid); Middle Left: Physidae; Middle Right: Planorbidae (some planorbid snails we collected were significantly larger than the one shown); Bottom Left: Valvatidae; Bottom Right: Viviparidae (most collected viviparid snails were too large to fit in the cell culture plate wells and looked more like the inset photo; the main photo is of a very young snail). [file peerj-12-17211-s002.pdf]
