# Supplementary material for: Freshwater trematodes differ from marine trematodes in patterns connected with division of labor
Source: PeerJ. 2024 Apr 12;12:e17211. doi: 10.7717/peerj.17211 (PMC11017974; doi:10.7717/peerj.17211)
Supplement: Supplemental Information 3 — Snails were identified to family (Snail Family) and were collected during several visits to each site (NMP, SP, TP, WR; see text for site locations) from late May through August. Reported is the total number of snails collected from each family (N Collected), the number of each snail family collected from each site (NMP, SP, TP, WR), the total number of infections from the collected snails (N Infected) and the number of infected snails whose rediae were included in the division of labor study (N Included). A few additional infections were included in the analysis from North Montpelier Pond (NMP) in October 2019 and summer 2021, but their collection data are not included in this table. [file peerj-12-17211-s003.pdf]

Supplementary File 3: Summary of the snails collected during summer 2019 including numbers of infections. Snails were identified to family (Snail Family) and were collected during several visits to each site (NMP, SP, TP, WR; see text for site locations) from late May through August. Reported is the total number of snails collected from each family (N Collected), the number of each snail family collected from each site (NMP, SP, TP, WR), the total number of infections from the collected snails (N Infected) and the number of infected snails whose rediae were included in the division of labor study (N Included). A few additional infections were included in the analysis from North Montpelier Pond (NMP) in October 2019 and summer 2021, but their collection data are not included in this table.

Snail Family	N Collected	NMP	SP	TP	WR	N Infected	N Included
Hydrobiidae	722	17	644	61	0	105	9
Lymnaeidae	29	0	7	16	6	0	0
Physidae	999	0	254	504	241	68	11
Planorbidae	859	45	203	360	251	81	32
Valvatidae	240	1	239	0	0	16	0
Viviparidae	655	530	0	125	0	20	2
Total	3496	585	1347	1066	498	292	54
