# Supplementary material for: Freshwater trematodes differ from marine trematodes in patterns connected with division of labor
Source: PeerJ. 2024 Apr 12;12:e17211. doi: 10.7717/peerj.17211 (PMC11017974; doi:10.7717/peerj.17211)
Supplement: Supplemental Information 5 [file peerj-12-17211-s005.docx]

**Supplemental File 5**: Description of column headings found in Supplemental File 4. Column headings are:

- sample: unique code assigned to each infection when it was collected. The letter at the start of the code indicates the family of snail from which the infection was isolated: H = Hydrobiidae, P = Planorbidae, Ph = Physidae, V = Viviparidae
- group: unique code assigned to each genetic group/COI species. Genetic groups are defined by 95% sequence similarity of the Folmer region of COI (sequenced using primers designed by VanSteenkiste et al. 2015).
- GenBank: accession number for NCBI GenBank COI sequence
- notes: notes describing why three of these infections were excluded from analysis
- coll.date: date the infected snail was collected in the form YYYYMMDD.
- coll.location: location from which the infected snail was collected. Abbreviations are NMP- North Montpelier Pond (44°18'21.9"N 72°26'46.7"W), SP- Shelburne Pond (44°22'37.8"N 73°09'43.2"W), TP- Ticklenaked Pond (44°11'31.1"N 72°05'49.7"W), WR- Waterbury Reservoir (44°22'54.8"N 72°43'36.0"W)
- dissect.medium: the dissection medium used to dissect the snail from which this colony was isolated. Media are DI water (w) or 0.1% saline (s).
- vol.N: the number of redia included in the analysis of whether volume is bimodal. The same set of photos were also used to look at reproductive potential (repr.) appendages (app.), pharynx size (phar., though not all rediae had pharynx size measured), proportion small (sm.), and distribution among different snail body regions (dist.).
- vol.SW.p: p-value from Shapiro-Wilk test for normality on volume measurements
- vol.hist.bimod: asks whether volume appears bimodal on the histogram. Results were only recorded for infections that showed a significant deviation from a normal distribution (vol.SW.p < 0.05)
- vol.hist.break: identifies where, i.e. what value of log(volume), the break between the two peaks in volume occurs on the histogram of volume. Recorded only if the distribution appears bimodal.
- repr.sm5: the maximum presence score for embryos received by any of the five smallest rediae examined for this infection. Presence score is on a scale of 0 to 4, with 0 indicating that embryos are definitely not present and 4 indicating that they definitely are. (Intermediate scores reflect uncertainty due to imperfect photos).
- app.ant.sm.median/app.ant.lg.median/app.post.sm.median/app.post.lg.median: median presence score for anterior (ant) or posterior (post) appendages, averaged among the largest (lg) or smallest (sm) 5 rediae examined for this infection. Presence scores are summarized above (repr.sm5)
- app.ant.p/app.post.p: p-value from a Kruskal Wallace Rank Sums test testing whether anterior (ant) or posterior (post) appendages were more prominent on small rediae.
- phar.N.lg/phar.N.sm: number of large (lg) or small (sm) rediae with measured pharynges. Classification as large vs. small depends on which peak of a bimodal histogram of volume the redia’s body size falls in or, for infections without bimodal volume, which quartile they fall in (small = quartile 1, large = quartile 4).
- phar.rel.median.big/phar.rel.median.sm/phar.abs.median.big/phar.abs.median.sm: median pharynx volume (abs; in μ^3^) or relative pharynx volume (rel; relative to body volume) of large (big) vs. small (sm) rediae.
- phar.abs.p/phar.rel.p: p-value from Kruskal Wallace Rank Sums test testing whether absolute (abs) or relative (rel) pharynx size differs between large vs. small rediae.
- sm.n.small/sm.n.big: number of rediae classified as small (small) vs. large (big). Size classification is described above (phar.N.lg/phar.N.sm).
- sm.prop.small: proportion of rediae with classified size (i.e. excluding any with intermediate size) that were classified as ‘small’. Size classification is described above (phar.N.lg/phar.N.sm)
- dist.gonad.n/dist.GonadMid.n/dist.mid.n/dist.midFoot.n/dist.foot.n/dist.general.n: the number of rediae isolated from the gonad, a combination of the gonad and mid-section (GonadMid; separation was not possible for some infections), mid section, a combination of the mid-section and head/foot (midFoot; again, if not separated), head-foot region (foot) or from any body section if the body sections were not separated (general).
- dist.gonad.p/dist.GonadMid.p/dist.mid.p/dist.midFoot.p/dist.foot.p/dist.general.p: the proportion of large and small rediae isolated from each body section (see above for descriptions) that were small
- dist.summary: a summary of whether the proportion of rediae that were small was higher in the head-foot (or mid section if none were found in the foot) than in the gonad (Y = yes, N = no)
- dist.CIsummary: a summary of whether the confidence intervals on the proportion of rediae that were small overlap between the head-foot (or mid-section) and gonad.
- act.abs.sm.med/act.abs.lg.med: median distance traveled by small (sm) vs. large (lg) rediae in two seconds, measured in mm.
- act.abs.p: p-value from Kruskal Wallace Rank Sum Test to determine whether there was a difference in the distance travelled by small vs. large rediae in 2 seconds.
- act.rel.sm.med/act.rel.lg.med: relative distance travelled by small (sm) vs. large (lg) rediae in two seconds; distance is relative to body length.
- act.rel.p: p-value from Kruskal Wallace Rank Sum Test to determine whether there was a difference in the relative distance travelled by small vs. large rediae in 2 seconds.
- att.lg/att.sm: number of attacks observed by large vs. small rediae
- att.what: description of what was attacked (e.g. same-species rediae, different-species cercaria)
- large.phar: a summary of whether rediae with unusually large pharynges were noticed in each infection
