# Supplementary material for: Freshwater trematodes differ from marine trematodes in patterns connected with division of labor
Source: PeerJ. 2024 Apr 12;12:e17211. doi: 10.7717/peerj.17211 (PMC11017974; doi:10.7717/peerj.17211)
Supplement: Supplemental Information 6 — If the genetic group includes trematodes from multiple infected snails, all snail numbers are listed in parentheses following the group number. For groups with many representatives, the snail number(s) from which photos were obtained are bolded. Scale bars are all 100 µm. [file peerj-12-17211-s006.pdf]

Supplementary File 6: representative photos of cercariae and rediae from each genetic group. If the genetic group includes trematodes from multiple infected snails, all snail numbers are listed in parentheses following the group number. For groups with many representatives, the snail number(s) from which photos were obtained are bolded. Scale bars are all 100µm.

Group	Cercaria	Smaller Redia	Larger Redia
H070 (H070, 110)	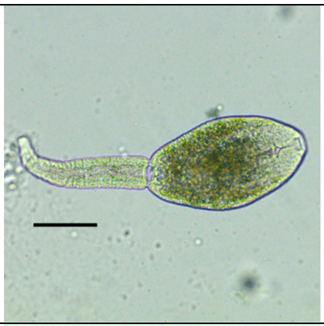	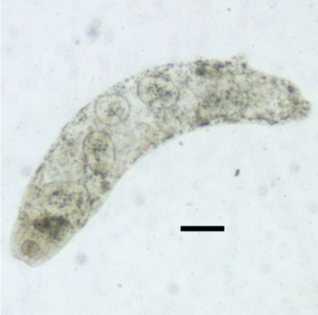	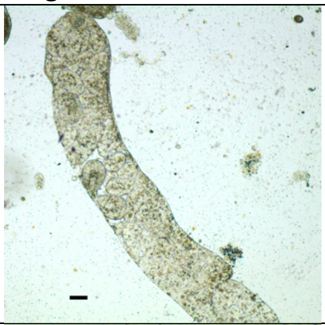
H102	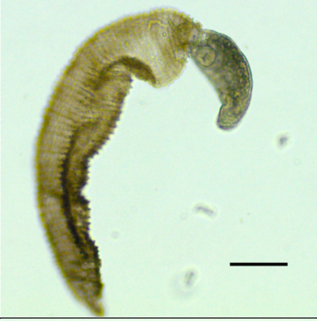	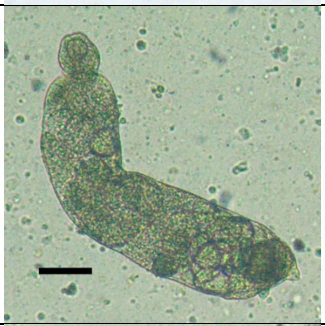	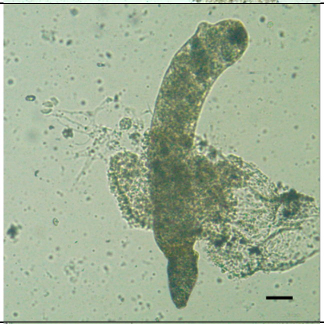
H104 (cercaria has finfold on tail; large redia are all slightly pinched/ damaged)	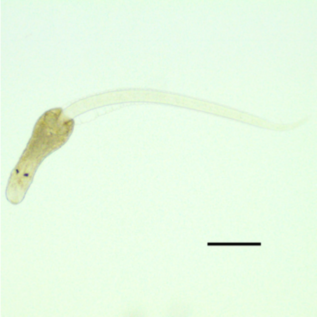	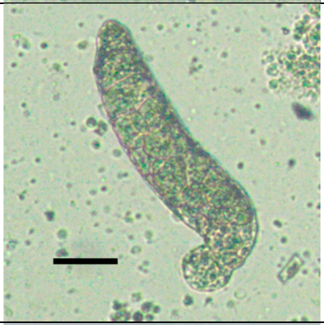	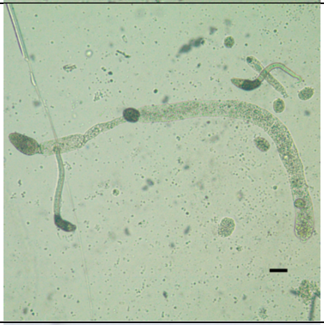
H107 (cercaria encysted before photo was taken; detached tail visible)	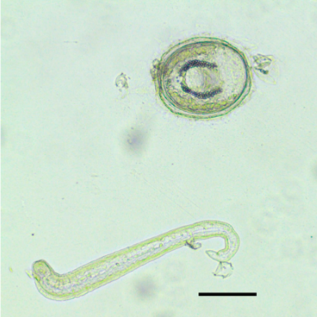	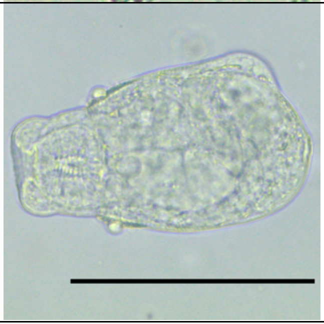	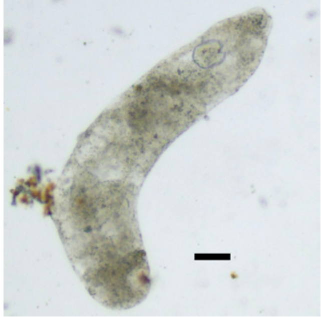

H127 (cercaria encysted before photo could be taken)	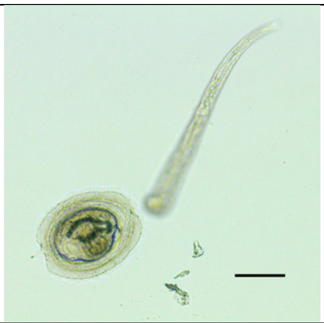	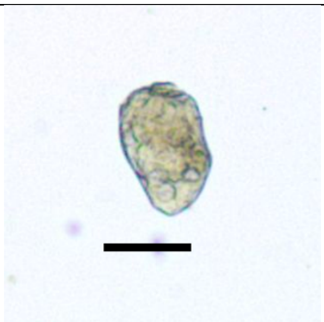	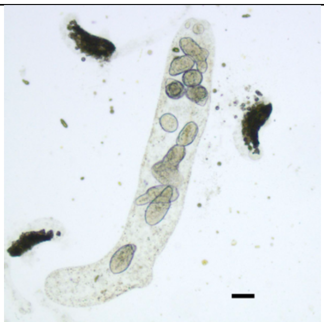
P107 (**P107**, P152)	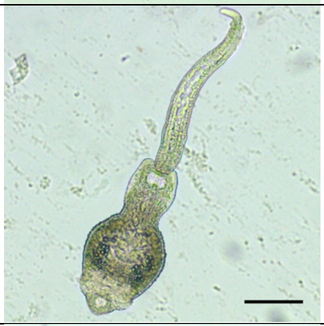	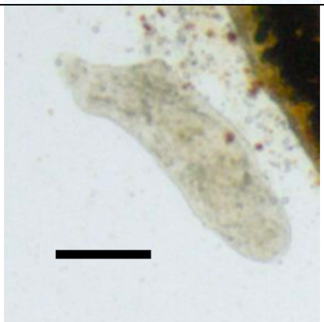	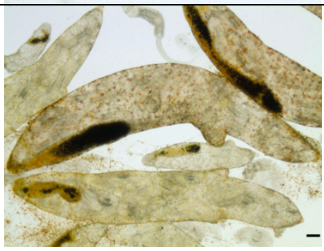
P112 (P119, P121, P122, P125, P126, P128, P131, **P137**, P154, P155, P160, P162, P163, P164, P165, P170)	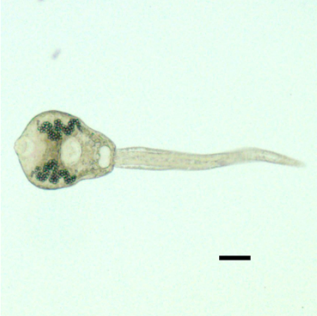	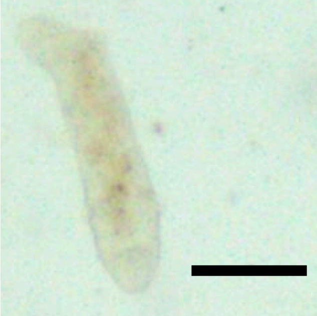	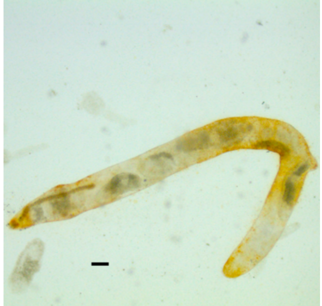
P113 (P113, **P120**, P130, **P136**, P157, P166)	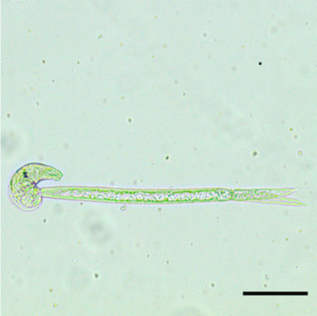	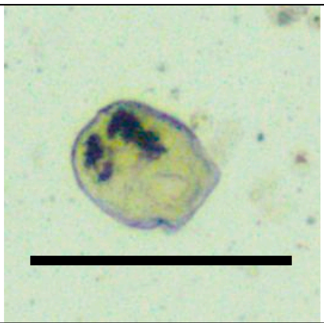	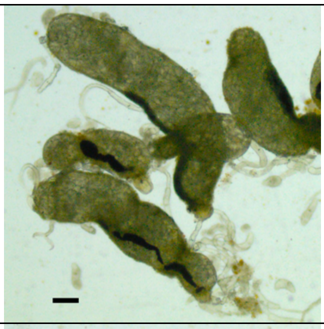
P115 (P115, P116, P117, **P140**, Ph126)	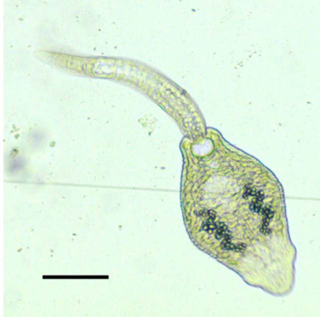	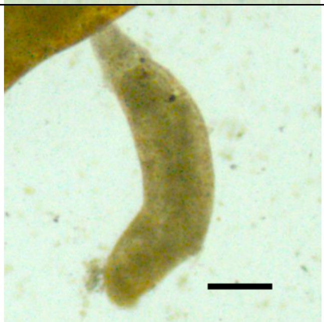	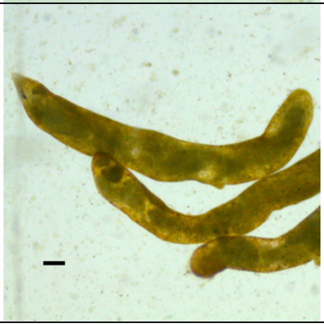

P141	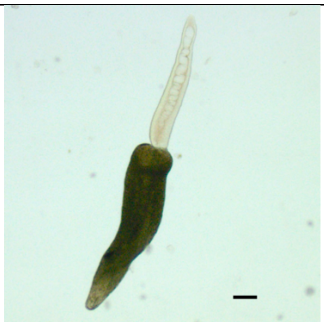	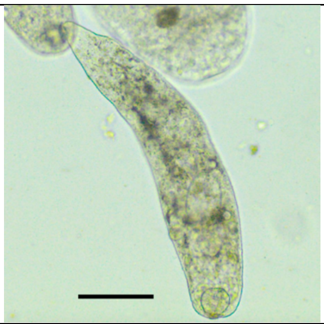	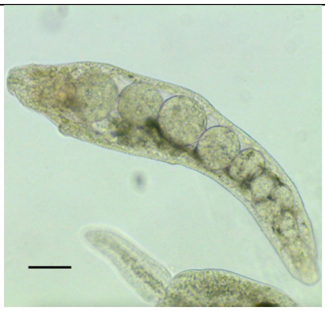
P150	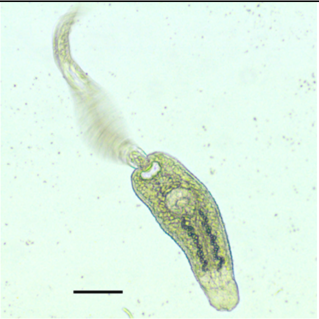	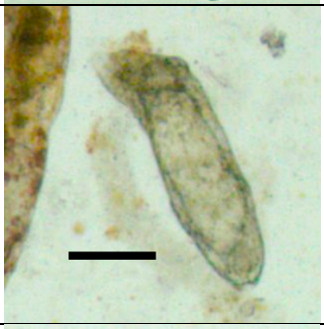	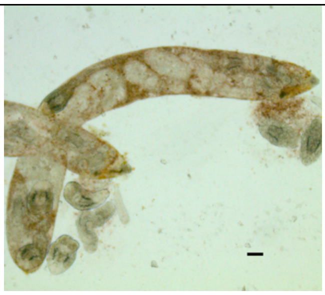
P153	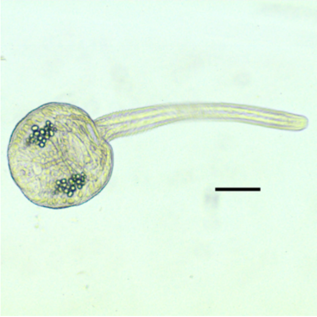	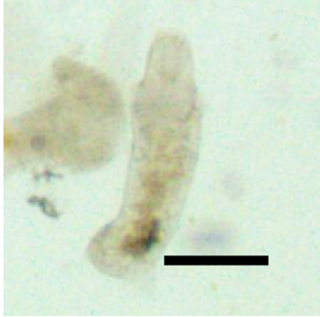	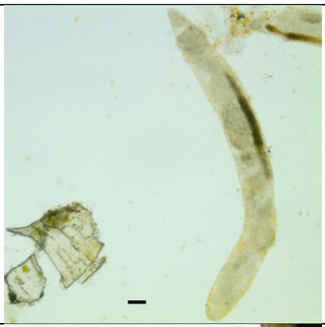
P177 (P177, P178)	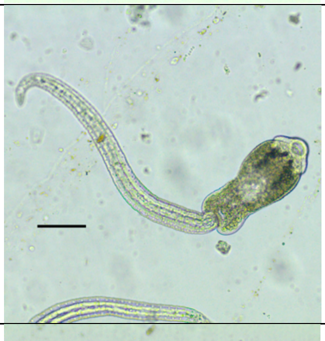	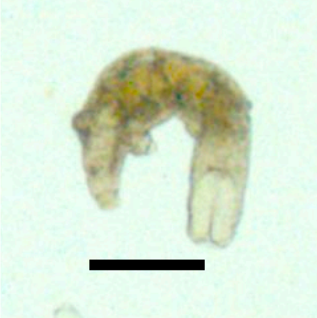	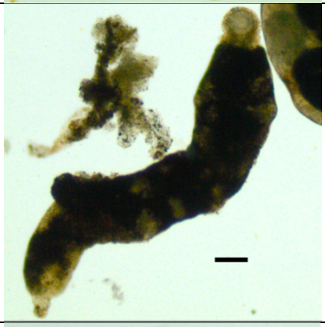
Ph095 (Ph095, **Ph107**, Ph109, Ph112)	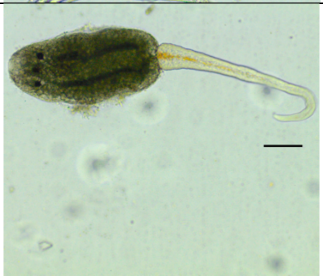	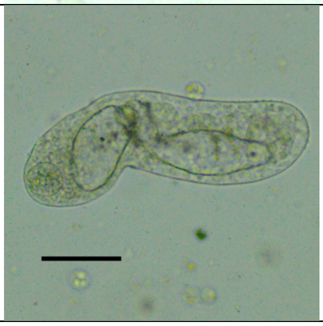	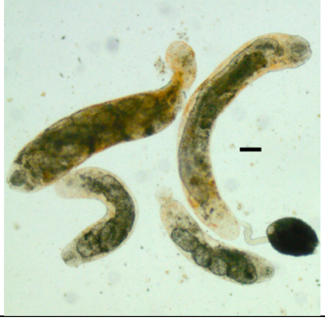

Ph096	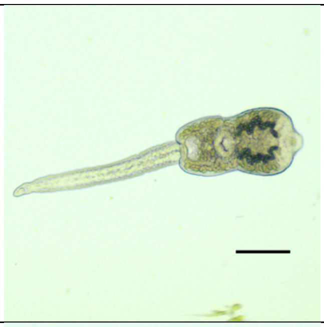	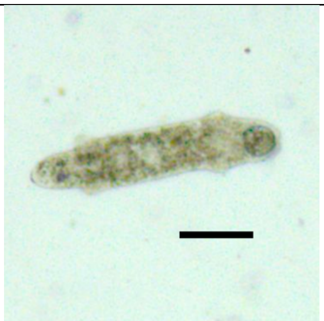	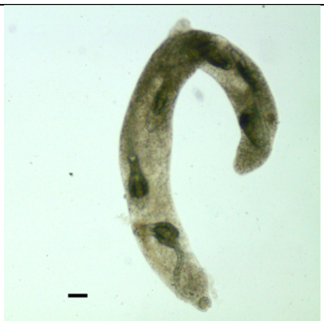
Ph100	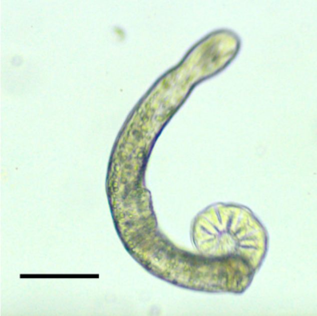	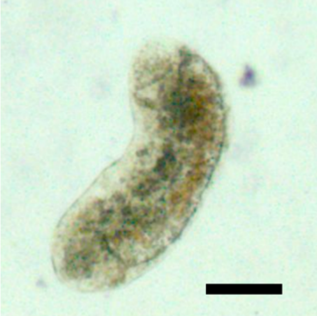	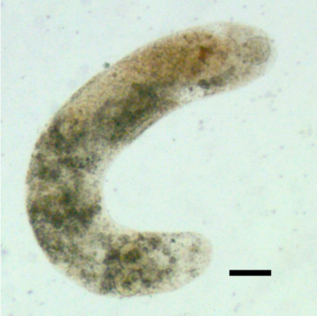
Ph116	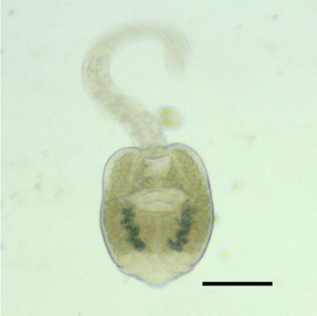	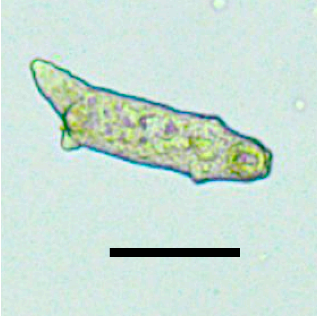	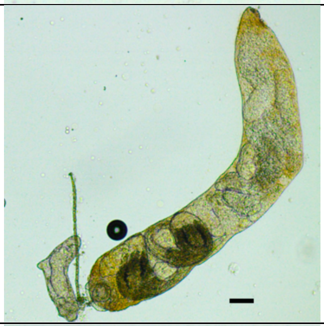
Ph159	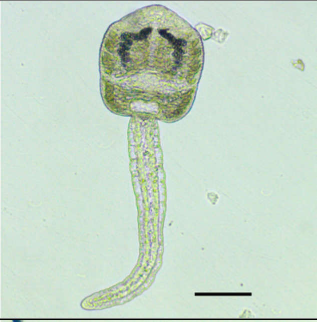	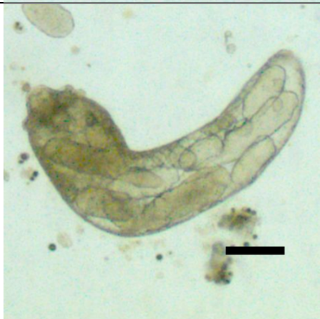	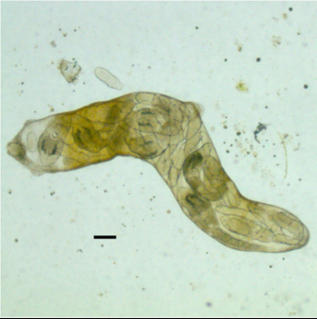
V141 (**V141**, V159, **V168**, V182, V183, V184)	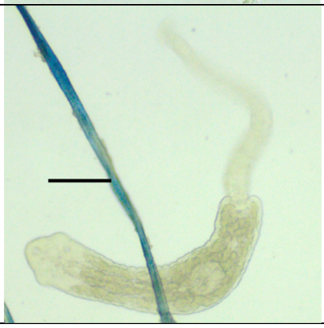	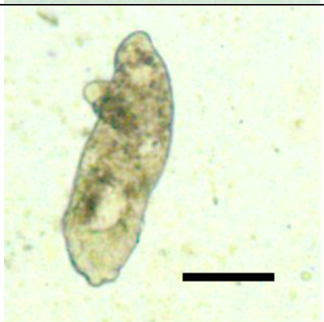	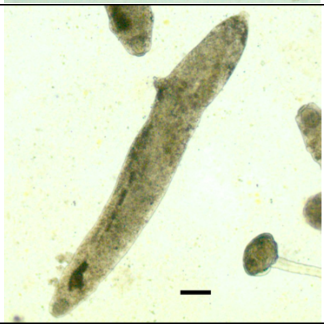

V172	(cercariae not observed before dissection; rediae were discovered after dissection)	(haphazard sample not taken; small may have been missed)	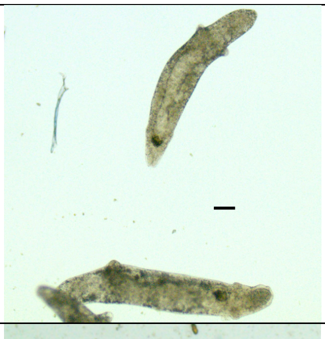
Unk: H148	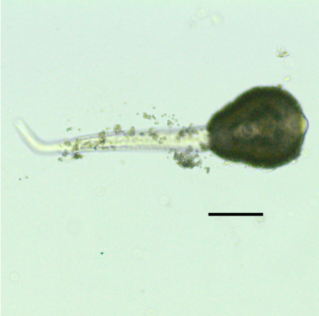	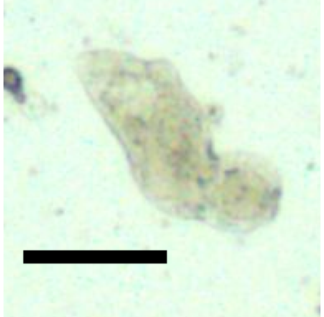	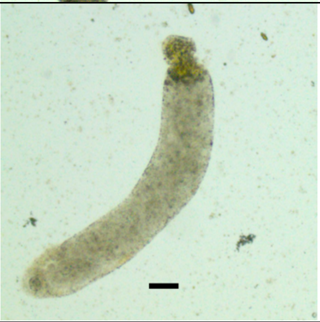
Unk: H158	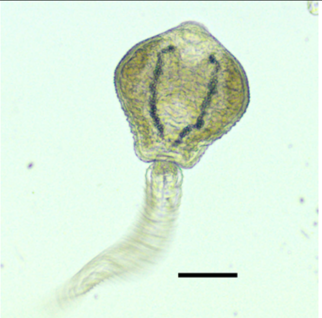	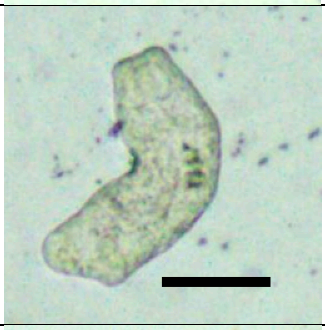	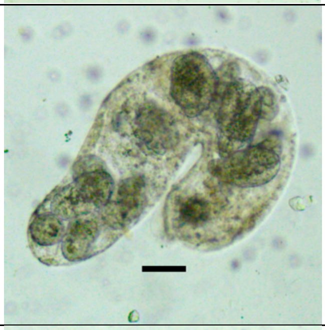
Unk: H096 (encysted before photo could be taken)	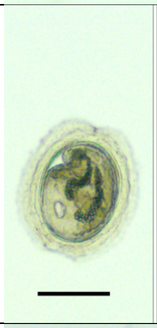 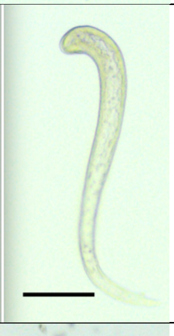	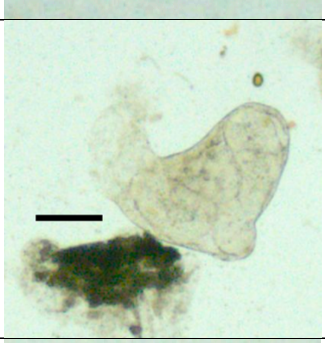	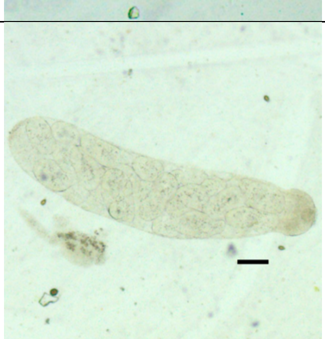
Unk: P135	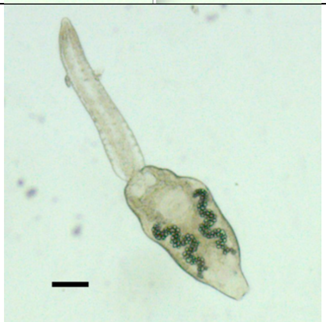	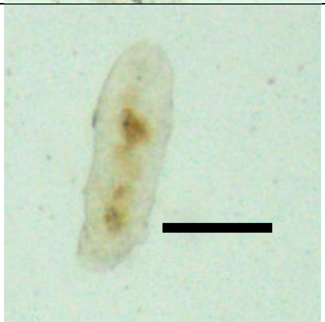	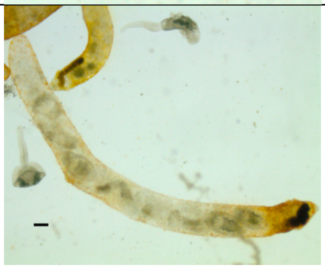

Unk: P139	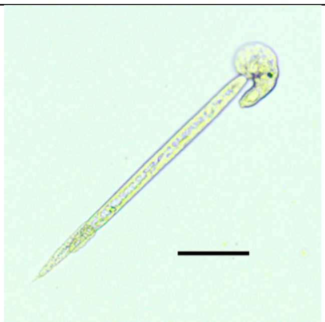	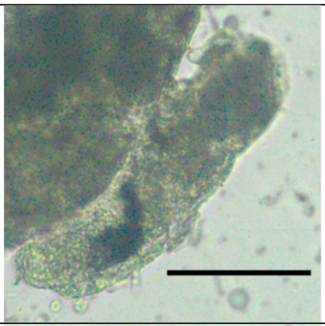	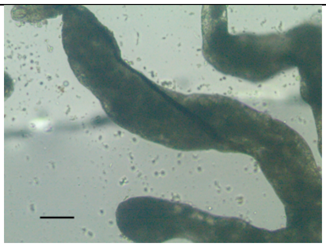
Unk: Ph115	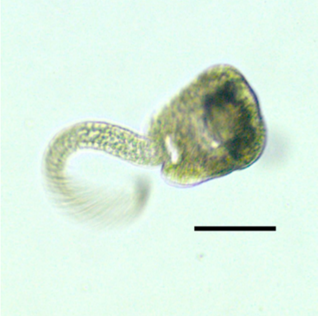	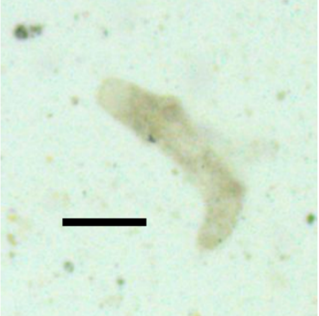	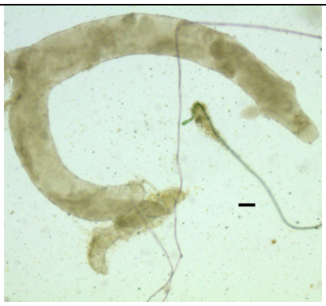
