# Supplementary material for: Freshwater trematodes differ from marine trematodes in patterns connected with division of labor
Source: PeerJ. 2024 Apr 12;12:e17211. doi: 10.7717/peerj.17211 (PMC11017974; doi:10.7717/peerj.17211)
Supplement: Supplemental Information 7 — For all panels, the rediae from individual wells of a cell culture plate are shown separately. Rediae were cultured with snail cells (circles and solid lines) or without snail cells (triangles and dashed lines) in one of three media: a 1:1 mixture of Bge Medium to Medium F (red), a 3:1 mixture of Bge Medium to Medium F (yellow), or Bge Medium only (teal). We stopped counting rediae in wells without snail cells after day 33 because at that point, all rediae appeared dead. Cercaria production was tracked for the first few timepoints, but it became increasingly difficult to count cercariae over time as tissues from dead cercariae started to break down (esp. in wells without snail cells) or snail cells became increasingly dense (in wells with snail cells). (A) shows the number of large “adult” rediae over time. Fluctuations in redia counts are probably counting error, not actual changes in the redia population size because it is a closed system and small rediae did not grow into large rediae during the timeframe monitored. Wells were initially seeded with 100μL of rediae, which corresponded to about 7–22 rediae per well and accounts for the differences in redia numbers among wells. (B) shows an estimate of the survival of rediae over time, measured as the percentage of rediae that were active while being observed for 5 s. Counts of active rediae were divided by the number of redia the well started with to allow easier visual comparison among wells. (C) shows the number of small redia that appeared in some of the wells over the course of the experiment. We assume that these small redia were produced by the larger rediae in those wells. [file peerj-12-17211-s007.pdf]

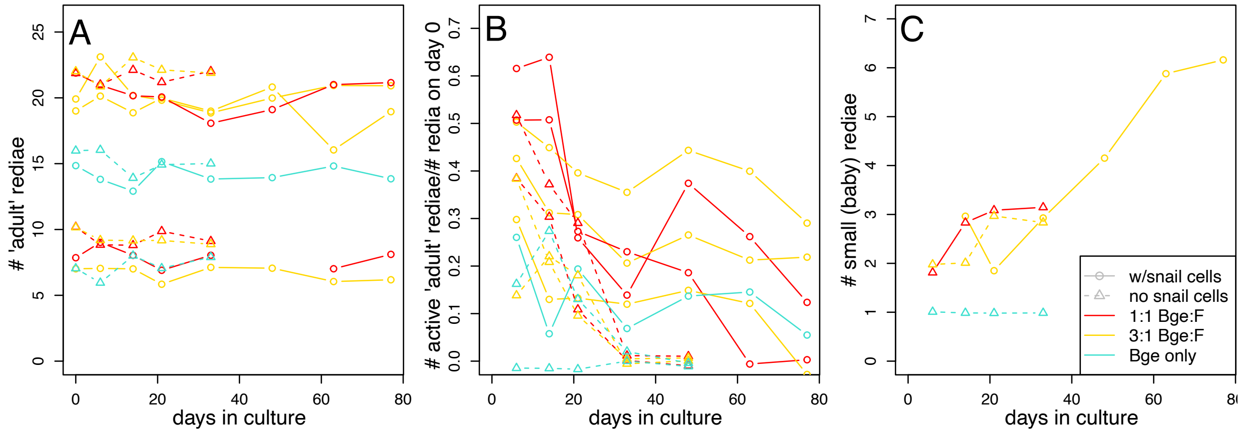

Supplementary File 7: Summary of redia counts, survival and reproduction in culture. For all panels, the rediae from individual wells of a cell culture plate are shown separately. Rediae were cultured with snail cells (circles and solid lines) or without snail cells (triangles and dashed lines) in one of three media: a 1:1 mixture of Bge Medium to Medium F (red), a 3:1 mixture of Bge Medium to Medium F (yellow), or Bge Medium only (teal). We stopped counting rediae in wells without snail cells after day 33 because at that point, all rediae appeared dead. Cercaria production was tracked for the first few timepoints, but it became increasingly difficult to count cercariae over time as tissues from dead cercariae started to break down (esp. in wells without snail cells) or snail cells became increasingly dense (in wells with snail cells). (A) shows the number of large “adult” rediae over time. Fluctuations in redia counts are probably counting error, not actual changes in the redia population size because it is a closed system and small rediae did not grow into large rediae during the timeframe monitored. Wells were initially seeded with 100µL of rediae, which corresponded to about 7-22 rediae per well and accounts for the differences in redia numbers among wells. (B) shows an estimate of the survival of rediae over time, measured as the percentage of rediae that were active while being observed for 5 seconds. Counts of active rediae were divided by the number of redia the well started with to allow easier visual comparison among wells. (C) shows the number of small redia that appeared in some of the wells over the course of the experiment. We assume that these small redia were produced by the larger rediae in those wells.
